# Intact lipid imaging of mouse brain samples: MALDI, nanoparticle-laser desorption ionization, and 40 keV argon cluster secondary ion mass spectrometry

**DOI:** 10.1007/s00216-016-9812-5

**Published:** 2016-08-22

**Authors:** Amir Saeid Mohammadi, Nhu T.N. Phan, John S. Fletcher, Andrew G. Ewing

**Affiliations:** 1 Department of Chemistry and Chemical Engineering, Chalmers University of Technology, Kemivägen 10, 41296 Gothenburg, Sweden; 2 National Center Imaging Mass Spectrometry, Kemivägen 10, 41296 Gothenburg, Sweden; 3 Department of Chemistry and Molecular Biology, University of Gothenburg, Kemivägen 10, 41296 Gothenburg, Sweden

**Keywords:** Mass spectrometry imaging, Gas cluster SIMS, MALDI, Nanoparticle-LDI, Lipids

## Abstract

We have investigated the capability of nanoparticle-assisted laser desorption ionization mass spectrometry (NP-LDI MS), matrix-assisted laser desorption ionization (MALDI) MS, and gas cluster ion beam secondary ion mass spectrometry (GCIB SIMS) to provide maximum information available in lipid analysis and imaging of mouse brain tissue. The use of Au nanoparticles deposited as a matrix for NP-LDI MS is compared to MALDI and SIMS analysis of mouse brain tissue and allows selective detection and imaging of groups of lipid molecular ion species localizing in the white matter differently from those observed using conventional MALDI with improved imaging potential. We demonstrate that high-energy (40 keV) GCIB SIMS can act as a semi-soft ionization method to extend the useful mass range of SIMS imaging to analyze and image intact lipids in biological samples, closing the gap between conventional SIMS and MALDI techniques. The GCIB SIMS allowed the detection of more intact lipid compounds in the mouse brain compared to MALDI with regular organic matrices. The 40 keV GCIB SIMS also produced peaks observed in the NP-LDI analysis, and these peaks were strongly enhanced in intensity by exposure of the sample to trifluororacetic acid (TFA) vapor prior to analysis. These MS techniques for imaging of different types of lipids create a potential overlap and cross point that can enhance the information for imaging lipids in biological tissue sections.

**Electronic supplementary material:**

The online version of this article (doi:10.1007/s00216-016-9812-5) contains supplementary material, which is available to authorized users.

**Graphical abstract:**
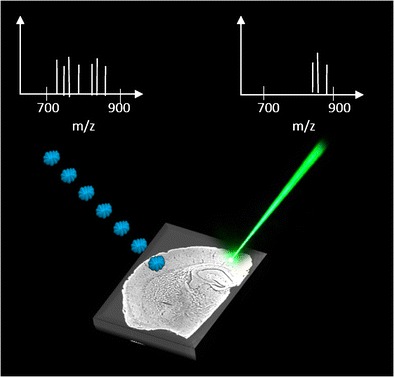
Schematic of mass spectral imaging of a mouse brain tissue using GCIB-SIMS and MALDI techniques

## Introduction

Lipids are one of the most abundant biomolecules in the brain with almost 60 % of the brain’s dry weight consisting of lipids. Lipids have a variety of biological functions and are the main constituents of cell membranes. In addition, they function as secondary messengers for cellular signal transduction across biological membranes, and they are involved in anchoring proteins within the plasma membrane. Cholesterol, sphingolipids (sphingomyelin, cerebrosides, sulfatides, gangliosides), and glycerophospholipids (phosphatidycholine, phosphatidylethanolamine, phosphatidylinositols) are three major categories of lipid compounds in the brain. The significant role of lipids in cell signaling and tissue physiology has been revealed by several studies on neurological disorders such as Alzheimer’s, Parkinson’s, Niemann-Pick, and multiple sclerosis which all show associated lipid alteration in the central nervous system [1–3].

Matrix-assisted laser desorption ionization (MALDI) and secondary ion mass spectrometry (SIMS) are two of the most widely used mass spectrometric imaging (MSI) techniques allowing direct probing of the distribution and composition of various lipid compounds in biological samples in a non-targeted and label-free manner [4, 5]. For instance, biochemical images of lipid distributions in single cells have been obtained using SIMS [6, 7], and both SIMS and MALDI are capable of showing the distribution of lipids in tissue [8–10].

MALDI has been primarily applied for analyzing intact lipids and higher mass compounds including proteins in biological samples [11, 12]. In MALDI, the sample is coated with a layer of organic matrix, which directly absorbs the laser energy and is ejected carrying the analyte molecules. The matrix removes energy from the analyte, reducing fragmentation. Predominantly intact analyte compounds are therefore produced by this soft ionization. Several methods of matrix deposition for MALDI imaging are commonly used including automated spraying (wet matrix deposition provides good extraction) and sublimation (produces small matrix crystals) [9, 13, 14]. These methods provide good ionization of the analyte while avoiding large crystal formation that can limit the spatial resolution with some wet matrix deposition techniques [15]. In addition, many organic matrices are only suitable for detection of larger molecules often with *m*/*z* >400 due to the interfering mass peaks derived from the organic matrix below this mass range [16]. Hence, detection of low mass ions by MALDI can be difficult. Low mass specific matrices such as carbon nanotubes [17], graphite, ionic liquids [18], and some high mass molecules [19] or use of matrix additives are capable of reducing background peaks in the low *m*/*z* region [20, 21].

Laser desorption ionization (LDI) [16] has recently enjoyed a resurgence by using nanostructured surfaces such as porous silicon as a support for chemical detection. Ionization and desorption of the sample are induced by UV laser irradiation of the surface called desorption-ionization on silicon (DIOS) mass spectrometry [22]. Nanoparticle-assisted LDI (NP-LDI) has been used as an alternative to the organic matrix for analyzing and imaging biomolecules such as fatty acids in tissue improving the ability to detect low mass species and produce less complex mass spectra compared with the standard organic matrices [23–25].

SIMS has been used to identify and localize different lipid species [26, 27]. The SIMS method has the ability to map chemical compounds with higher lateral resolution compared to the other MSI techniques. An advantage of SIMS imaging is that there is no specific sample pretreatment needed. However, extensive fragmentation of molecular species is a disadvantage that makes SIMS best suited for detection of low mass species *m*/*z* <1000. Gas cluster ion beams (GCIBs) are a relatively new primary ion source for SIMS [28] and have emerged as a potential tool to improve the detection limit of SIMS for analyzing intact lipids compared to the conventional cluster (Bi_3_^+^) and even polyatomic primary ion beams (C_60_^+^) [29–31]. There are benefits suggested for argon cluster SIMS of organic samples such as high sputter yield, higher yield for high mass secondary ions, and less subsurface chemical damage compared to conventional primary ion sources. It has been recognized that Ar cluster primary ions may lead to a softer ejection process so that higher mass molecular ions can be desorbed intact from the surface [29]. Recently, a continuous high-energy Ar cluster ion beam (40 keV Ar_4000_^+^) has been used to investigate the potential benefits for lipid analysis and imaging in fly and mouse brains [32, 33].

Conventional SIMS is a hard ionization technique making it difficult to image the intact molecular ions of lipids. In contrast, MALDI and NP-LDI have been used to identify and image lipid molecular ions in biological tissues. Gold nanoparticles have been used in lipid molecular ion imaging and compared with the 2,5-dihydroxybenzoic acid (DHB) matrix using MALDI-ion mobility in which gold nanoparticles allowed detection of different peaks compared to DHB MALDI [34]. This motivates us to investigate and compare conventional MALDI and NP-LDI to the newly developed high-energy gas cluster primary ion SIMS to image intact lipids.

In this paper, we examine and compare the newly developed high-energy Ar cluster SIMS to both NP-LDI and MALDI for the detection of intact lipids and imaging in mouse brain sections. In order to demonstrate the complementary analysis in the MSI, lipid molecular ions detected by our optimized gold NP-LDI [35] and conventional MALDI have been assigned and compared to those from the 40 keV gas cluster ion beam secondary ion mass spectrometry (GCIB SIMS). The combined approach extends the number of lipid species detected by mass spectrometry imaging techniques in mouse brain samples. Moreover, we demonstrated the possibility of Ar GCIB in focusing to sub-5 μm providing the ability to detect the extended number of molecular lipid species and maximize the information obtained from lipids with higher spatial resolution than conventional MALDI and nanoparticle-assisted laser desorption ionization mass spectrometry (NP-LDI MS). We show that the 40 keV GCIB helps to maximize the information obtained from lipid imaging and is beneficial for high spatial resolution imaging of lipid pseudo-molecular ions in mouse brain.

## Experimental section

### Tissue preparation

**Fig. 1:**
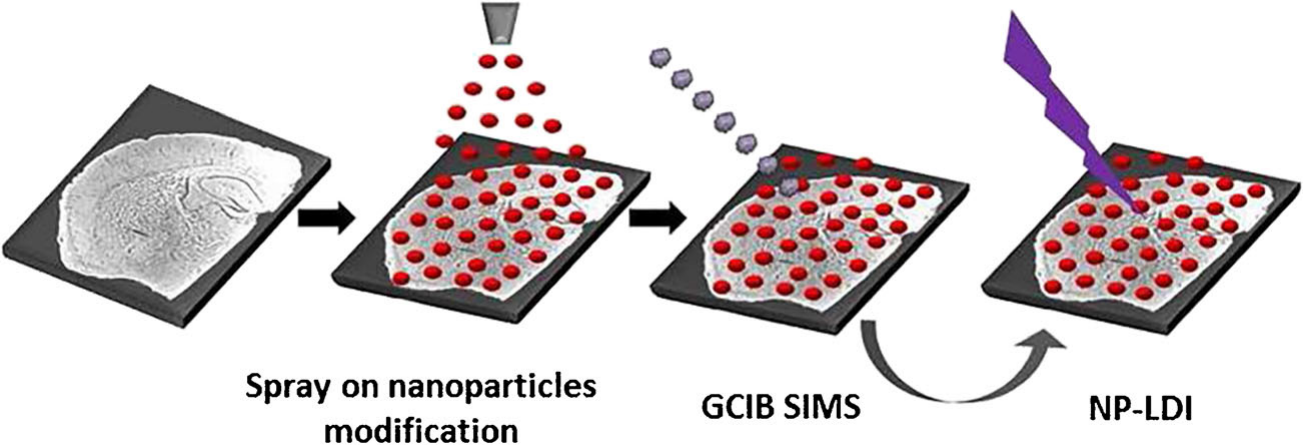
Workflow for tissue imaging using SIMS and NP-LDI. A mouse brain tissue section after freeze-drying was modified with Au nanoparticles using a spray on procedure. The modified sample was then subjected to SIMS and subsequently to NP-LDI imaging

Frozen mouse brain tissue was cryo-sectioned (Cryostat Leica CM1520) at −20 °C and sagittal brain slices with 12 μm thickness were thaw mounted on indium tin oxide (ITO)-coated glass microscope slides. Slides were placed on a cooled (approximately −20 °C) stainless steel holder in a vacuum desiccator and gradually brought to the room temperature under vacuum for approximately 1 h. Tissue sections were modified with Au nanoparticles using an airbrush for NP-LDI analysis. The 10-nm citrate-capped gold nanoparticles (prepared using the citrate reduction method described by Kimling et al. [36]) suspended in ethanol at 0.6 mg/mL concentration were sprayed at a distance of 15 cm from the tissue to avoid wetting the sample [35]. The same Au NP-modified section was analyzed sequentially by GCIB SIMS and NP-LDI (Fig. 1). Different tissues were used for positive and negative ion modes. For MALDI experiments, serial tissue sections from the same brain were collected and DHB (Sigma-Aldrich, Stockholm, Sweden) matrix was applied by sublimation according to the procedure described in our previous study [37]. Briefly, DHB was sublimed at temperature 145 °C under pressure set to 0.8 mbar for total time 10 min.

### Mass spectrometric imaging and data analysis

SIMS imaging was carried out using the J105—3D Chemical Imager (Ionoptika Ltd, Southampton, UK) equipped with a 40 keV gas cluster ion beam producing Ar_4000_^+^ (8 % CO_2_ in Ar). The continuous stream of secondary ions that are generated are pulsed into a quadratic field reflectron time-of-flight analyzer using a linear buncher decoupling the MS from the ion generation process [38, 39]. A sample of 1200 × 1200 μm^2^ area was analyzed in positive ion mode with a 20-μm beam step (pixel size) and a primary ion fluence of 1.05 × 10^12^ ions/cm^2^.

An Ultraflextreme MALDI ToF/ToF mass spectrometer (Bruker Daltonics, Bremen, Germany) instrument was used for NP-LDI and MALDI analysis. Imaging data were acquired by summing 500 shots per array position (pixel) at the laser repetition rate of 1000 Hz. Both NP-LDI and MALDI imaging were performed in positive ion mode with 20-μm laser step intervals (pixel size). Profiling of Au NP-modified brain tissue using NP-LDI was carried out in negative ion mode and also for investigation of trifluororacetic acid (TFA) sample modification with NP-LDI. Mass spectra for profiling were obtained by summing up 1000 shots at a laser repetition rate of 1000 Hz per spot.

TFA modification of the sample was carried as previously described [40]. Briefly, the tissue slide was placed at 1-cm distance from a glass petri dish containing 300 μL of TFA solution 99 % in a sealed desiccator. The tissue was exposed for 30 min at room temperature.

## Results and discussion

We compare NP-LDI, MALDI, and GCIB SIMS to investigate some of the diverse lipid classes present in regions of mouse brain tissue. Studying lipids with these MSI techniques provides complementary spectral and distributional information of intact lipids in the mouse brain, which can maximize the available information when tackling biological questions.

### Nanoparticle-modified mouse brain sample is compatible with 40 keV GCIB SIMS analysis

A sample section coated with 10-nm Au nanoparticles was used (a scanning electron microscopy image confirming the homogeneity of nanoparticle coverage is shown in the Electronic Supplementary Material (ESM) Fig. S1) for both SIMS and LDI analysis. SIMS does not normally need any sample surface modification, although several studies have reported increased secondary ion signals when using matrix-enhanced SIMS (ME-SIMS) and metal-assisted SIMS (MA-SIMS) [41–43]. Previously, we showed that nanoparticle modification is compatible with SIMS analysis on a standard POPC lipid compound sample [35]. Mouse brain sections, however, contain a variety of lipid species. Nanoparticle-free brain, as a control, and nanoparticle-modified brain sections were analyzed by SIMS using the Ar_4000_^+^ GCIB, and the spectra were compared to investigate the compatibility of this sample preparation with SIMS. The spectra of control and nanoparticle-modified brains are similar, although there is a moderate enhancement (twofold) in the secondary ion yield in the modified brain. The result demonstrates that nanoparticle modification is therefore compatible with SIMS analysis and does not induce any observable lipid chemical change in the *m*/*z* 700–900 range for the mouse brain sample (ESM Fig. S2). Likewise, Ar cluster SIMS analysis prior to the NP-LDI experiment does not make a difference in the detected peaks using NP-LDI (ESM Fig. S3).

### Detection of intact lipid species in the mouse brain using NP-LDI, MALDI, and GCIB SIMS

Nanoparticle modification is an alternative to the organic matrix normally used in MALDI [24, 34, 44]. Using the nanoparticles eliminates background in the low mass range that can arise from the organic matrix in MALDI. Furthermore, the same Au nanoparticle-modified mouse brain section can be analyzed with both Ar cluster SIMS and NP-LDI techniques to obtain reliable comparisons and complementary lipid information. To compare to the MALDI technique where organic matrices are used, we analyzed a subsequent tissue slice from the same mouse brain with sublimed DHB matrix and MALDI. Sublimation was chosen as the matrix application method as the small crystals formed are more comparable with the NP addition, and one of the overall goals of MALDI is to push the spatial resolution to the sub-cellular scale (even though a 20-μm pixel size was selected for this study). Furthermore, advantages of MALDI matrix sublimation over the spraying methods such as increased signal, minimized lateral diffusion of lipids, and generation of a uniform thin layer of matrix have been shown by Robert Murphy, particularly for imaging of lipids in tissue slices [45]. The Caprioli group has also demonstrated improvements in spatial resolution in MALDI MS imaging employing transmission geometry laser optics, and in this case sublimation of matrix was also used [46].

**Fig. 2:**
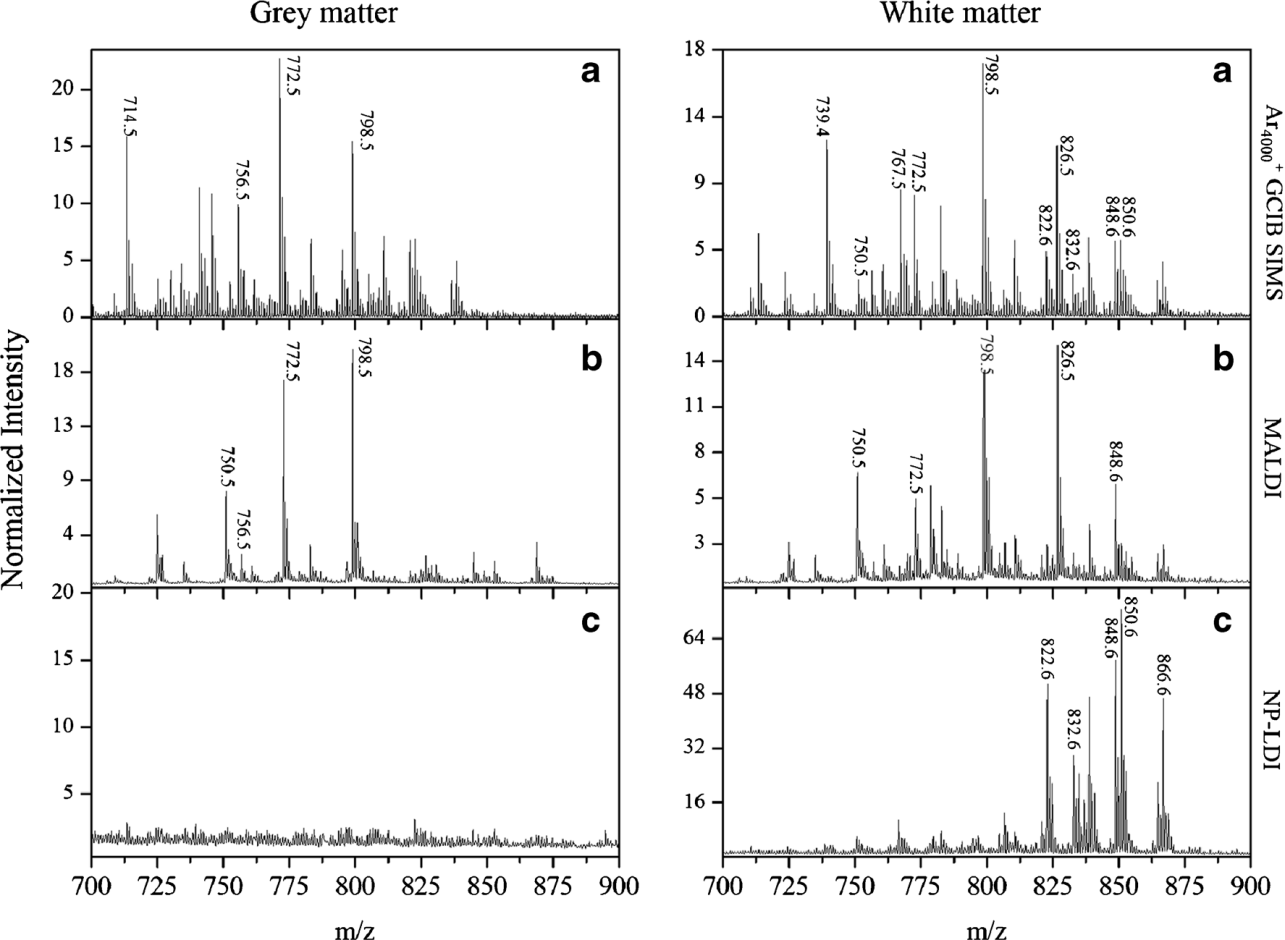
Comparison of the mass spectra in the gray and white matter regions obtained from a brain tissue section within the intact lipid mass range (*m*/*z* 700–900) using **a** 40 keV Ar_4000_^+^ GCIB SIMS on the nanoparticle-modified tissue, **b** MALDI with DHB sublimation, and **c** gold NP-LDI. The spectra for all methods were normalized to total ion counts in the whole spectrum (In SIMS ×10^9^ for gray matter and ×10^4^ for white matter)

Intact lipids in the mass range *m*/*z* 700–900 were discretely imaged in white and gray matter areas of the brain sample with three approaches; MALDI, NP-LDI, and SIMS. For NP-LDI and SIMS, these were done on the same sample in the two instruments sequentially. Spectra in the mass range *m*/*z* 150–900 from both gray and white matter are presented in ESM Fig. S4. Although SIMS is usually considered a harder ionization technique producing low mass lipid fragments, comparison of the intact lipid region of the mass spectra of the high-energy cluster SIMS and MALDI (Fig. 2a versus b) demonstrates that improved ion yields for high mass molecular species (intact lipid ions) are obtained with the cluster SIMS. High mass intact lipid peaks similar to MALDI are obtained, and furthermore, the GCIB SIMS shows additional peaks compared to MALDI when considering the intact lipid mass spectra in both gray and white matter regions of brain. NP-LDI analysis clearly results in different peaks from the similar brain section analyzed by DHB MALDI analysis, a phenomenon that was also observed previously [34, 37, 47, 48]. However, in Fig. 2c, it is clear that the molecular ion peaks obtained using NP-LDI originate from lipid compounds localized specifically in the white matter, and there are no peaks detected from the gray matter area. Peaks acquired by NP-LDI are observed in the GCIB SIMS mass spectra analyzed from the same sample section. For the mouse brain samples, GCIB SIMS can be used to analyze and identify the combination of ion peaks detected by MALDI and NP-LDI indicating the potential of this approach as a promising technique for imaging larger and intact lipid species in the brain.

Assignment of the peaks, based on the literature and online databases, reveals that 40 keV GCIB SIMS and MALDI mass spectra are dominated by strong peaks from lipids phosphatidylcholine (PC) (the base peak in the presented spectra corresponds to POPC, [M + K]^+^, *m*/*z* 798.54), in contrast to NP-LDI, which mainly produces signals from lipids with *m*/*z* >800 such as galactosylceramide and sphingomyelin species and does not produce an intact POPC signal. The NP-LDI result is consistent with our previous work in that NP-LDI analysis on POPC standard lipid does not show the intact molecular peak for POPC but instead its fragments are detected (*m*/*z* 184.07 (C_5_H_15_NPO_4_) and *m*/*z* 86.09 (C_5_H_12_N)) [35]. In NP-LDI analysis of the brain, it appears that POPC (and probably other intact PCs) is strongly fragmented by the laser with nanoparticles and thus its molecular ion and most PCs with *m*/*z* <800 do not appear in the NP-LDI mass spectrum. Therefore, NP-LDI might be a suitable method for selective analysis of lipid species in the brain, particularly for those lipids distributing in the white matter area. This especially includes lipids such as cerebroside and sphingomyelin (with *m*/*z* above 800), which are usually covered by the cholesterol in the white matter and can hardly be detected in SIMS.

The different ionization processes that occur when using MALDI with an organic matrix versus NP-LDI might be used to explain the diverse peaks detected and different mass spectra. The organic matrix is a low volatility chemical that absorbs energy from the laser radiation and indirectly couples the energy to the analyte co-crystallized with the matrix. In NP-LDI, the mechanism for analyte ionization/desorption is a thermally driven process. In this mechanism, the excitation of gold nanoparticles can produce heat due to the interactions between electrons in the particles and laser irradiation. The resulting heat is then transferred to the analytes. In complex biological sample matrices, the thermal propagation to different molecules is different, and therefore the ionization efficiency is different. This results in different signal responses of biomolecules and the various mass spectra obtained from different samples. The complete fragmentation of the PC lipids to *m*/*z* 184.07 and smaller fragments with the NP-LDI compared with partial fragmentation in the GCIB SIMS experiment provide an indication as to the relative energetics of the ion formation processes.

### Trifluoroacetic acid sample modification for NP-LDI and GCIB SIMS in positive ion mode

**Fig. 3:**
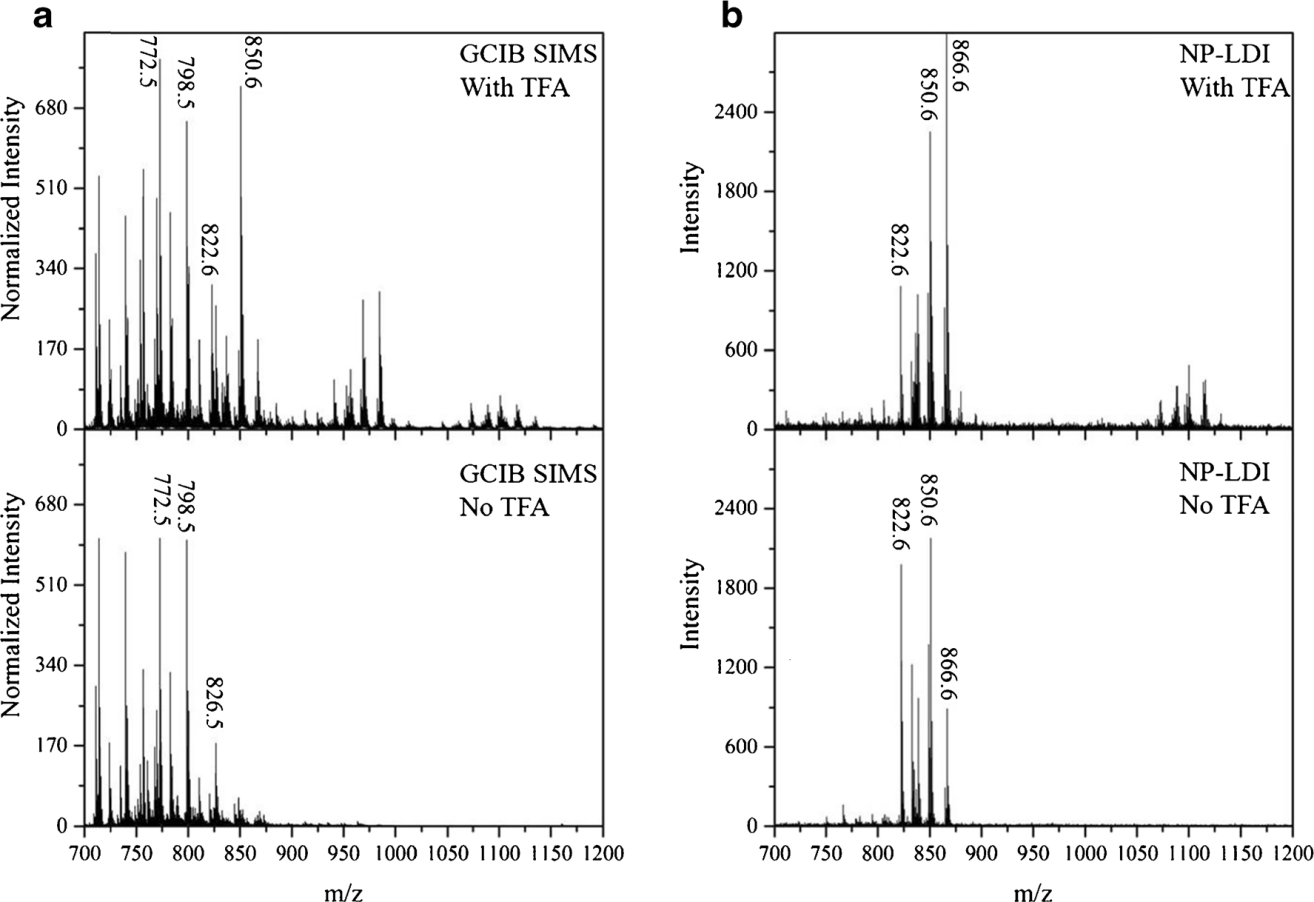
Mass spectra of the TFA treated sample (*top*) and without TFA exposure (*bottom*) using Ar cluster SIMS (**a**) in comparison with NP-LDI analysis (**b**). The spectra for SIMS methods were normalized to number of selected pixels

In contrast to MALDI, the SIMS technique usually does not need as much sample preparation. However, to improve the ionization ability of higher mass molecules, there are methods to treat the sample prior to the SIMS analysis such as organic matrices (matrix enhancement, ME-SIMS) or metals (metal assisted, MA-SIMS) coating [41]. TFA has been commonly used as a component in MALDI matrix solution and also in desorption electrospray ionization (DESI) studies [49]. Recently, it was shown that when using SIMS analysis (on mouse brain) or DHB sublimation, MALDI (on fly brain) sample modification with TFA vapor exposure is beneficial for the measurement of intact lipids since it helps to remove cholesterol in cholesterol-rich samples, and it might slightly improve the lipid signals by enhancing ionization [37, 40]. We applied the same method of TFA treatment to compare GCIB SIMS, NP-LDI, and MALDI with DHB analysis when examining positive ions. In our experiment on the mouse brain, TFA treatment results in no significant change in lipid intensities observed using DHB sublimation for MALDI. For NP-LDI analysis, sample modification with TFA increases the intensities of intact lipid ions in the range *m*/*z* 800–900 and additionally it produces new peaks above *m*/*z* 1000, which are not detected without the TFA modification (Fig. 3). In comparison, when the GCIB is used for SIMS, the peak intensities are enhanced significantly, especially for *m*/*z* >800 including the lipid region where NP-LDI produces the most peaks. As the peaks detected by NP-LDI are only observed in the white matter of the mouse brain sample, we hypothesize that the TFA removes cholesterol from the surface of the white matter allowing the other species to be measured with SIMS. Comparison of the peak at *m*/*z* 369 belonging to (cholesterol + H-H_2_O) between control and TFA-treated samples indicates that cholesterol content is diminished by applying TFA (ESM Fig. S5). This is consistent with the mechanism proposed by Angerer et al. previously [40].

### Molecular ion imaging of phospholipid species in the mouse brain

**Fig. 4:**
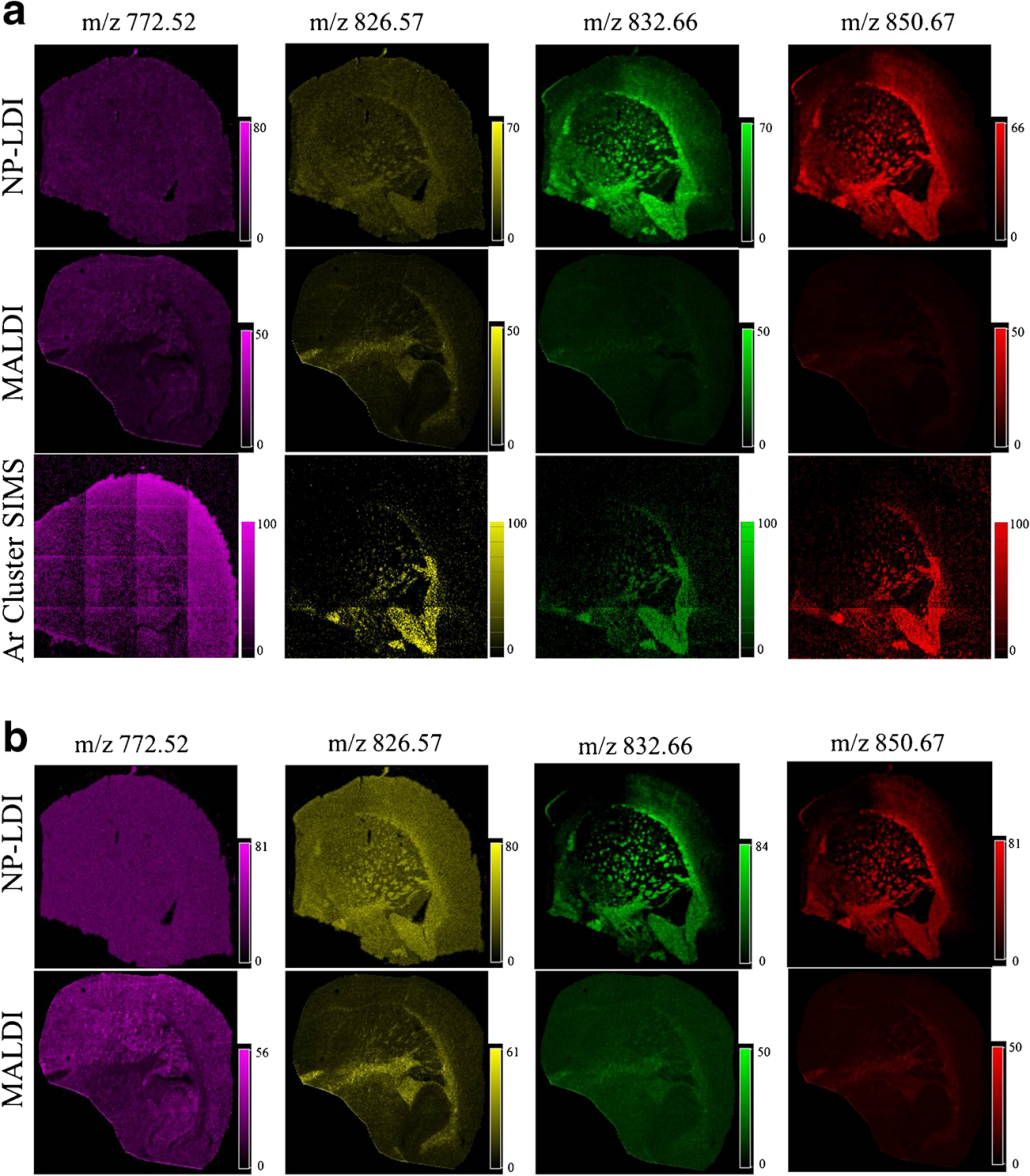
Ion images (no normalization (**a**) and total ion count (*TIC*) normalization (**b**) (4800 × 4800 μm^2^)) obtained from mouse brain tissue using NP-LDI, MALDI with 20-μm laser step size, and Ar GCIB SIMS adjusted to have 20-μm beam spot size. Dominant peaks at *m*/*z* 832.66 (sphingolipid [M + Na]^+^) and *m*/*z* 850.67 (cerebroside C_48_H_93_NO_9_ [M + Na]^+^) were selected for NP-LDI for comparison with dominant peaks at *m*/*z* 772.52 (PC (32:0), [M + K]^+^) and *m*/*z* 826.57 (PC (36:1), [M + K]^+^) for MALDI and Ar GCIB SIMS. NP-LDI images demonstrate distinct distributions for lipid species in the white matter while this is not distinguishable for MALDI with DHB. Ar GCIB SIMS shows the ions localized in both white and gray matter. The number of counts in Ar GCIB SIMS for *m*/*z* 772.52, *m*/*z* 826.57, *m*/*z* 832.66, and *m*/*z* 850.67 are 3709, 1763, 1069, and 1176, respectively

To highlight the differences between detected peaks with these MSI techniques, two peaks in the mass spectra from each method NP-LDI (*m*/*z* 832.66 and 850.67) and MALDI (*m*/*z* 772.52 and 826.57) were selected (four peaks in total) and their ion images compared with the same peaks in the Ar GCIB SIMS mass spectrum without any signal normalization (Fig. 4a, b). As shown above, in NP-LDI the intact lipids are only localized in the white matter, we see the sodium adduct of sphingolipid (C_48_H_91_NO_8_Na) at *m*/*z* 832.66 and sphingomyelin (18:1/24:1) at *m*/*z* 835.66. Sphingomyelins are the most abundant sphingolipids in mammalian cell membrane; they are the main components of the myelin sheath that acts as an isolation layer around the axons of myelinated neurons. Besides being components of cell membranes, sphingomyelins act as regulators for various cellular signaling processes such as cell growth, signal transduction, and apoptosis [50]. In addition, they are abundant in high-density lipoproteins, and sphingomyelins affect protein conformation and can therefore regulate the activities of enzymes, receptors, and transporters [51]. In the white and gray matter of human brain, the sphingomyelin content is about 7–8 % in each area, whereas in whole rat brain, the content is about 4 % [52]. Previously, MALDI imaging of the mouse brain was used to show that different sphingomyelins specifically localize in the gray matter or white matter corresponding to the localization of ceramide synthase, which is the enzyme that catalyzes the synthesis of the precursor of sphingomyelins—ceramides. For example, the sphingomyelins (18:1/24:0) and (18:1/24:1) localize in the white myelin-rich matter, whereas sphingomyelin (18:1/18:0) distributes in the gray matter [53].

The ion images show that the cerebroside (sodium adduct of C_48_H_93_NO_9_ at *m*/*z* 850.67) is observed by NP-LDI and is colocalized with the sphingomyelins (18:1/24:0) and (18:1/24:1) in the white matter (Fig. 4a). Cerebrosides are glycosphingolipids which are composed of a ceramide connected to a sugar unit [54]. There are two groups of cerebrosides, which are the glucocerebrosides (glucosylceramides) and galatocerebrosides (galatosylceramides). Glucosylceramides are mainly found in the cytoplasmic surface of the cell membrane, skin, muscle, intestine, and kidney [55, 56], whereas galactosylceramides are typically present in the brain. Galactosylceramides are the abundant lipid components of the neuronal myelin sheath [57]. Cerebrosides function as anticoagulant in the cardiovasculature and in the brain, and are important regulators for cell differentiation, apoptosis, and signal transduction, for example galactosylceramides affect the activities of Ca channels and cell morphology [58, 59]. Thus, the ability to measure these species with high spatial resolution and reasonable sensitivity is highly important.

The ion images acquired by Au NP-LDI clearly demonstrate (without any data normalization) the distribution of detected lipids in the white matter with an enhanced contrast compared to MALDI with the same 20-μm pixel size using DHB sublimation which does not show any heterogeneity in the signal distribution in the ion images (Fig. 4a).

Therefore, to provide clear ion images, data normalization (versus total ion counts) has been applied to MALDI with DHB as matrix, and the ion images have been compared to the NP-LDI images using the same normalization (normalized data images are shown in Fig. 4b). Comparing the ion images shows that in contrast to the NP-LDI, the distribution of lipids in the gray matter, for instance the potassium adduct of PC (32:0) [M + K]^+^ ion with *m*/*z* 772.52, is more distinctive in MALDI. Likewise, the ion image of *m*/*z* 734.56 indicates the distribution of protonated peak for PC (32:0) in the gray matter is also more distinct in comparison with the potassium adduct of this lipid species in LDI (ESM Fig. S6).

For Ar GCIB SIMS, with the pixel size set to 20 μm for closer comparison with the MALDI and LDI data, the ion images of intact lipids without any data normalization clearly show the distribution of the analytes in both the gray, such as potassium adduct of PC (32:0) [M + K]^+^ ion, and white matter, such as potassium adducts of PC (36:1) [M + K]^+^, sphingolipid C_48_H_91_NO_8_ [M + Na]^+^, and cerebroside C_48_H_93_NO_9_ [M + Na]^+^ (Fig. 4a).

Phosphatidylcholines belong to the glycerophospholipids group which are the main components of the cell membrane. The lamellar-shaped PCs mainly distribute in the outer leaflet of the bilayers of the cell membrane. PCs have been shown to play important roles in brain function and perturbation of PCs leads to severe brain disorders and injuries, for instance Alzheimer’s disease, ischemia, stroke, and schizophrenia [60, 61]. In the rat brain, different PCs have been observed in different regions depending on the length and number of double bonds of fatty acid chains. For example, PC (32:0) at *m*/*z* 734 is predominantly localized in the gray matter of the rat brain, whereas PC (36:1) at *m*/*z* 788 is distributed in both gray and white matter [62, 63]. This correlates to our results, which show that potassium adduct PC (32:0) [M + K]^+^ is localized in the gray matter.

**Fig. 5:**
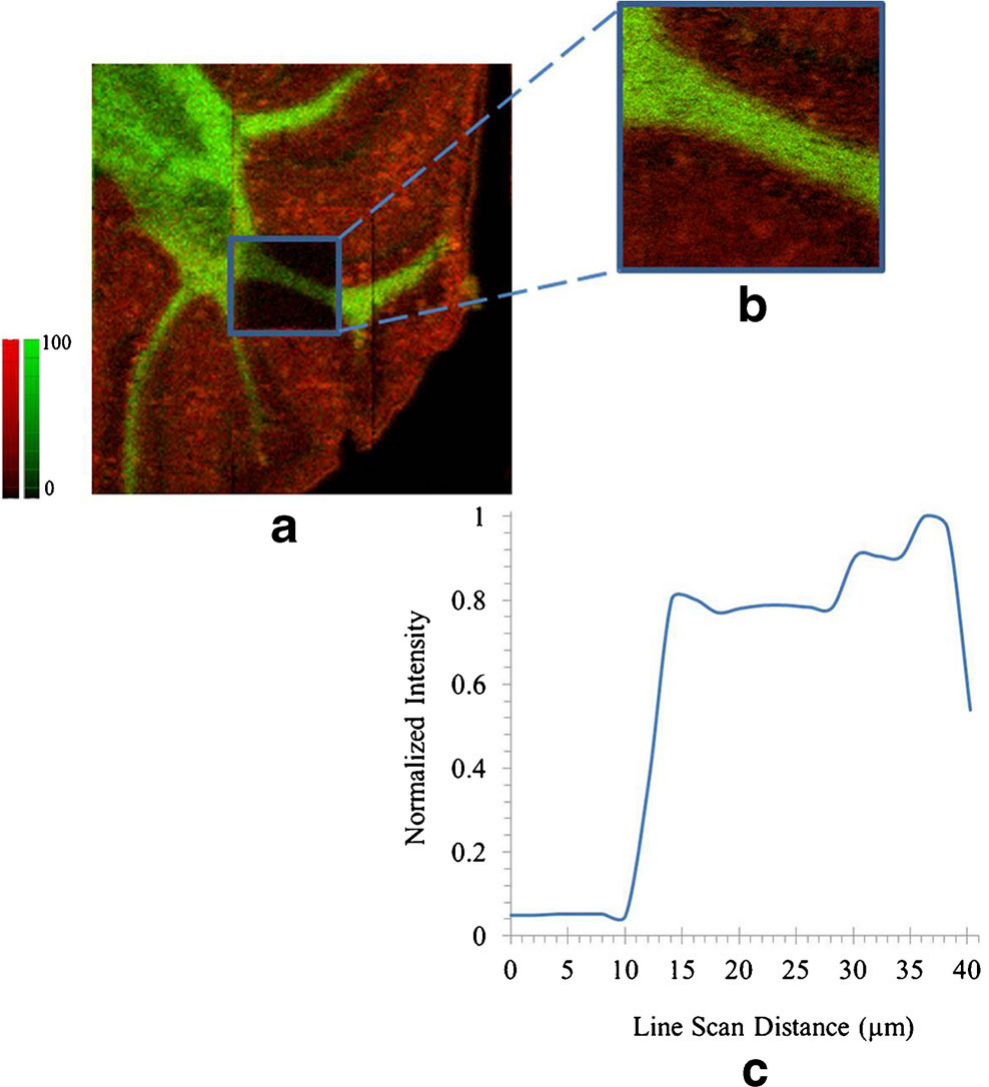
Overlay ion images of intact PC (32:0) [M + K]^+^ at *m*/*z* 772.52 (*red*) and sphingolipid C_48_H_91_NO_8_Na [M + Na]^+^ at *m*/*z* 832.66 (*green*). The pixel size was set to 12 μm for image (**a**) (2400 × 2400 μm^2^, with the number of counts 20,337 for *m*/*z* 772.52 and 15140 for *m*/*z* 832.66) and 3 μm for image (**b**) (500 × 500 μm^2^, with the number of counts 9525 for *m*/*z* 772.52 and 9040 for *m*/*z* 832.66). The line scan graph was obtained from image (**b**) indicates an approximate spatial resolution of 4 μm (**c**)

Utilizing the 40 kV Ar GCIB for biological imaging provides benefits including higher secondary ion signal for high molecular weight species which is beneficial for high spatial resolution imaging of intact molecular ions such as lipids. To demonstrate this point, we acquired an image of a TFA-modified mouse brain section using GCIB SIMS with smaller beam sizes. Images of intact lipids distributed in the white and gray matter are shown with pixel sizes of 12 and 3 μm in Fig. 5 (a and b, respectively). This is a promising advantage of using GCIB SIMS to perform high spatial resolution (approximately 4 μm) mapping of high mass intact lipids such as sphingomyelins, phosphatidylcholines and cerebrosides.

### Analysis using Ar GCIB SIMS and NP-LDI in negative ion mode

**Fig. 6:**
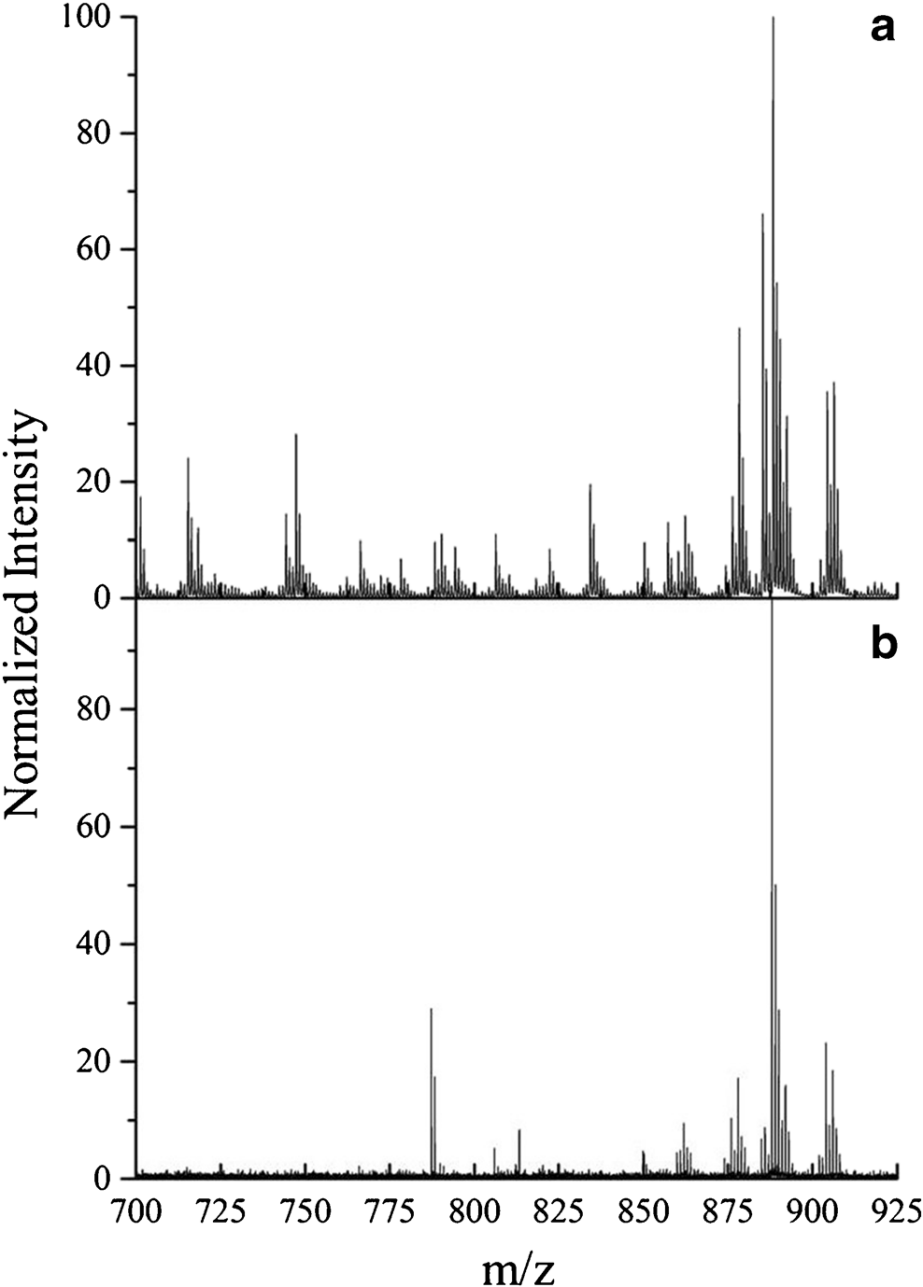
Mass spectra of intact lipids acquired from mouse brain tissue using high-energy Ar cluster SIMS (**a**) and NP-LDI (**b**) in negative ion mode (peak intensity is normalized to the maximum intensity (×100) in the range m/z 700 to m/z 925). The strong peak in the NP-LDI spectrum between *m*/*z* 775 and *m*/*z* 800 is Au_4_^−^

NP-LDI and SIMS allow the detection of further lipid species in the negative ion mode. There are also several methods that have been reported for negative ion lipid analysis including MALDI using organic matrixes such as α-cyano-4 hydroxycinnamic acid (CHCA), 9-aminoacridine, or 2,5-DHB [64, 65]. Figure 6 shows mass spectra for NP-LDI and high-energy Ar cluster SIMS in the negative ion mode containing intact lipid peaks in the range *m*/*z* 700–925. The spectrum for the high-energy Ar cluster SIMS shows that lipids in the negative ion mode can be detected similarly to those detected by applying organic matrix (DHB) with washing procedures (beneficial for peaks enhancement) in MALDI reported in previous studies [65]. Furthermore, in comparison to NP-LDI (Fig. 6), it is obvious that a lot more peaks are again observed in the intact lipid molecular range by Ar GCIB SIMS.

### Peak identification and related lipid compound assignment

Putative peak assignments, based on literature and database assignments, for mouse brain comparing intact molecular ions and lipid species identified by GCIB SIMS, NP-LDI, and MALDI in positive and negative ion modes are given in Tables 1 and 2. The peaks identified by each MSI technique are shown by a plus sign (+). The high-energy Ar_4000_^+^ GCIB can be applied to identify many of the lipid species observed with MALDI, but additionally it can be used to detect and image molecular species, for instance PC (34:0) [M + H]^+^, phosphatidylinositol PI (30:1) [M + H]^+^, or cerebroside C_40_H_76_NO_9_ [M + H]^+^, in the range of *m*/*z* 700–800 not easily observed with MALDI.

**Table 1:** Positive ion mode

Possible assignments for lipid molecular ion peaks	Calculated mass	Ar SIMS	MALDI	NP-LDI	Measured mass Ar SIMS	∆ppm
Galactosylceramide (32:1) C_38_H_73_NO_8_K [M + K]^+^	710.4973	+			710.495	−3.23
Phosphatidic acid (34:1) C_37_H_71_O_8_PK [M + K]^+^	713.4523	+			713.4537	1.96
Cerebroside C_40_H_76_NO_9_ [M + H]	714.5519	+			714.5553	4.75
Phosphatidic acid C_39_H_73_O_8_PNa [M + Na]^+^	723.494	+			723.4953	1.79
Phosphatidylcholine (32:0) C_39_H_73_O_8_PK [M + K]^+^	734.5699	+	+		734.5677	−2.99
Phosphatidic acid C_39_H_73_O_8_PK [M + K]^+^	739.4679	+			739.4683	0.54
Phosphatidic acid (36:1) C_39_H_75_O_8_PK [M + K]^+^	741.4836	+			741.4826	−1.34
Sphingolipid (18:0) C_42_H_81_NO_8_NA [M + Na]^+^	750.5859	**+**	+		750.5789	−9.32
Phosphatidylcholine fragment	751.52	+	+		751.5181	−2.52
Sphingomyelin (18:1/36:1) C_41_H_83_N_2_O_6_PNA [M + Na]^+^	753.5886	+			753.5873	−1.72
Phosphatidylcholine (32:0) C_40_H_80_NO_8_PNa [M + Na]^+^	756.5519	+	+		756.5541	2.9
Phosphatidylcholine (34:2) C_42_H_81_NO_8_ [M + H]^+^	758.5699	+			758.5634	−8.56
Phosphatidylcholine (34:1) C_42_H_83_NO_8_P [M + H]^+^	760.5856	+	+		760.5868	1.57
Phosphatidylcholine (34:0) C_42_H_85_NO_8_P [M + H]^+^	762.6012	+			762.5996	−2.09
Phosphatidylinositol (30:1) C_39_H_76_O_12_P [M + H]^+^	767.5074	+			767.5073	−0.13
Sphingomyelin (18:1/36:1)C_41_H_83_N_2_O_6_PK [M + K]^+^	769.5625	+			769.5614	−1.42
Phosphatidylcholine (32:0) C_40_H_80_NO_8_PK [M + K]^+^	772.5258	+	+		772.5251	−0.9
Sphingomyelin (18:1/20:0) C_43_H_87_N_2_O_6_PNa [M + Na]^+^	781.6199	+	+		781.6157	−5.37
Phosphatidylcholine (34:1) C_42_H_82_NO_8_PNa [M + Na]^+^	782.5675	+			782.5678	0.38
Phosphatidylcholine C_42_H_84_NO_8_PNa (34:0) [M + Na]^+^	784.5831	+			784.5833	0.25
Phosphatidylcholine (36:1) C_44_H_87_NO_8_P [M + H]^+^	788.6169	+			788.6164	−0.63
Phosphatidylcholine (34:1) C_42_H_82_NO_8_PK [M + K]^+^	798.5414	+	+		798.5426	1.5
Phosphatidylcholine (34:0) C_42_H_84_NO_8_PK [M + K]^+^	800.5571	+			800.554	−3.87
Sphingomyelin (18:1/22:0)C_45_H_91_N_2_O_6_PNa [M + Na]^+^	809.6512	+		+	809.6438	−9.13
Phosphatidylcholine (36:1) C_44_H_86_NO_8_PNa [M + Na]^+^	810.5988	+			810.5941	−5.79
Galactosylceramide (d18:1/23:0) C_47_H_91_NO_8_Na [M + Na]^+^	820.6642	**+**		+	820.6583	−7.18
Phosphatidylcholine (40:4) C_48_H_89_NO_7_P [M + H]^+^	822.6376	**+**		+	822.6394	2.18
Galactosylceramide (d18:0/22:0) C_46_H_91_NO_8_K [M + Na]^+^	824.6381	**+**		+	824.6315	−8
Phosphatidylcholine (36:1) C_44_H_86_NO_8_PK [M + K]^+^	826.5727	+	+		826.579	7.62
Sphingolipid C_48_H_91_NO_8_Na [M + Na]^+^	832.6642	**+**		+	832.658	−7.44
Sphingomyelin (18:1/24:1) C_47_H_93_N_2_O_6_PNa [M + Na]^+^	835.6668	**+**		+	835.6619	−5.86
Phosphatidylcholine (42:1) C_47_H_92_NO_7_PNa [M + Na]^+^	836.6508	**+**			836.654	3.82
Sphingomyelin (18:1/24:0)C_47_H_95_N_2_O_6_PNa [M + Na]^+^	837.6825	**+**		+	837.6744	−9.66
Phosphatidylethanolamine (42:3) C_47_H_88_NO_8_PNa [M + Na]	848.6144	+	+	+	848.619	5.42
Cerebroside C_48_H_93_NO_9_Na [M + Na]^+^	850.6747	**+**		+	850.668	−7.87
Sphingomyelin (18:1/24:1) C_47_H_93_N_2_O_6_PK [M + K]+	851.6408	**+**		+	851.6442	3.99
Phosphatidylcholine (41:4) C_49_H_91_NO_8_P [M + H]^+^	852.6482	**+**			852.6492	1.17
Ceramide (40:0) C_46_H_93_NO_11_P [M + H]^+^	866.6486	**+**		+	866.6437	−5.65

Lipid assignment for mass peaks obtained by NP-LDI, MALDI, and high-energy Ar cluster SIMS in the mouse brain in positive ion mode (Table 1) and peak assignments for NP-LDI and Ar GCIB SIMS spectra in negative ion mode (Table 2) [26, 40, 48, 66]. The peaks that appear in each MSI technique are weighted by (+). In Ar GCIB SIMS, the peaks enhanced after TFA modification of the sample are bold. Galactosylceramide (A/B) corresponds to long chain base (A)/ amide-linked fatty acid (B)

**Table 2:** Negative ion mode

Class	Calculated mass	Molecular species	Measured mass	∆ppm
Acidic glycosphingolipids/Sulfoglycosphingolipids [M-H]^-^	778.5138	C_40_H_76_SNO_11_	778.5156	2.31
	794.5088	C_40_H_76_SNO_12_	794.5123	4.4
	806.5451	C_42_H_80_SNO_11_	806.5457	0.74
	850.5739	C_44_H_84_SNO_12_	850.5726	−1.52
	862.6077	C_46_H_88_SNO_11_	862.6045	−5.24
	876.6234	C_47_H_90_SNO_11_	876.6188	−5.24
	878.6026	C_46_H_88_SNO_12_	878.6017	−1.02
	888.6234	C_48_H_90_SNO_11_	888.6231	−0.33
	890.639	C_48_H_92_SNO_11_	890.6386	−0.44
	904.6183	C_48_H_90_SNO_12_	904.6185	0.22
	906.6339	C_48_H_92_SNO_12_	906.6333	0.66
Glycerophosphoinositols/Diacylglycerophosphoionositols [M-H]^-^	857.5179	C_45_H_78_O_12_P	857.5169	−1.16
	885.5492	C_47_H_82_O_13_P	885.5485	−0.79

In negative ion mode, the most dominant peaks detected by both NP-LDI analysis and Ar cluster SIMS are shown. Measured mass obtained from Ar SIMS

A considerable number of molecular ion adduct peaks in the NP-LDI spectrum suggest an ability for the Au nanoparticles to generate cationized ions. Some lipid species in the brain such as sphingolipid C_48_H_91_NO_8_ [M + Na]^+^, sphingomyelin (18:1/24:1) [M + K]^+^, or cerebroside C_48_H_93_NO_9_ [M + Na]^+^ are dominantly identified and imaged by NP-LDI, whereas there are also sodium-cationized pseudo-molecular ion peaks produced by NP-LDI such as cerebroside C_46_H_89_NO_9_ [M + Na]^+^ (*m*/*z* 822.64) that are detected with their pronated or molecular ion peaks in the Ar GCIB SIMS and MALDI spectra. The NP-LDI dominant peaks are enhanced clearly by analysis with the GCIB SIMS after TFA modification of the sample. Moreover, in the negative ion mode, Ar GCIB SIMS also shows the capability of detecting a wider range of lipids with good signal to noise compared with NP-LDI. Besides lipid compounds including sphingolipids (acidic glycosphingolipids and sulfatides) and phospholipids that are similarly identified by NP-LDI and SIMS, other intact lipid peaks in the range *m*/*z* 700–875 are only observed using the Ar GCIB SIMS analysis (detailed peaks have been assigned in a previous study [40]). In fact, these results indicate that it is possible to use Ar GCIB SIMS imaging to analyze the wide range of intact lipid species detected in the mass range to about 900 *m*/*z* by both NP-LDI and MALDI from mouse brain samples.

## Conclusions

GCIB SIMS has been compared to both NP-LDI and MALDI mass spectrometric imaging techniques in terms of their ability to analyze and image intact lipids in mouse brain tissue. NP-LDI gives high signals in the mass range *m*/*z* >800 and groups of intact lipids localized in the white matter are selectively detected, whereas MALDI shows high sensitivity for phosphatidylcholine lipids in the mass range *m*/*z* <800. NP-LDI exhibits superior potential for ion imaging of the lipid species specifically localized in the white matter compared to MALDI with conventional organic matrix deposition. In MALDI, there is a need for data normalization to obtain more distinctive ion images. However, SIMS with a 40 keV Ar_4000_^+^ GCIB is capable of detecting intact lipid compounds present in both the white and gray matter areas of the mouse brain sample in both positive and negative ion modes similar to the lipid analysis by MALDI, and furthermore in positive ion mode it is possible to detect more lipid species in the *m*/*z* 700–900 range. In addition, the peaks detected by NP-LDI were significantly enhanced in the GCIB SIMS spectrum following TFA exposure. Ar GCIB SIMS provides the ability to detect a wide range of molecular lipids including those detected by MALDI and NP-LDI with a beam that is readily focusable to sub-5 μm which makes it an encouraging technique in biochemical imaging and lipidomics.

## Electronic supplementary material

Below is the link to the electronic supplementary material.

ESM 1 (PDF 1.17 mb)

## References

[1] Han X, Holtzman MD, McKeel WD, Kelley J, Morris JC (2002). Substantial sulfatide deficiency and ceramide elevation in very early Alzheimer’s disease: potential role in disease pathogenesis. J Neurochem.

[2] Hartmann T, Kuchenbecker J, Grimm MOW (2007). Alzheimer’s disease: the lipid connection. J Neurochem.

[3] Woods AS, Jackson SN (2006). Brain tissue lipidomics: direct probing using matrix-assisted laser desorption/ionization mass spectrometry. AAPS J.

[4] Caprioli RM, Farmer TB, Gile J (1997). Molecular imaging of biological samples: localization of peptides and proteins using MALDI-TOF MS. Anal Chem.

[5] Chaurand P, Schwartz SA, Caprioli RM (2002). Imaging mass spectrometry: a new tool to investigate the spatial organization of peptides and proteins in mammalian tissue sections. Curr Opin Chem Biol.

[6] Fletcher JS, Rabbani S, Henderson A, Lockyer NP, Vickerman JC (2011). Three-dimensional mass spectral imaging of HeLa-M cells—sample preparation, data interpretation and visualisation. Rapid Commun Mass Spectrom.

[7] Kurczy ME, Piehowski PD, Van Bell CT, Heien ML, Winograd N, Ewing AG (2010). Mass spectrometry imaging of mating tetrahymena show that changes in cell morphology regulate lipid domain formation. Proc Natl Acad Sci USA.

[8] Berry KAZ, Li B, Reynolds SD, Barkley RM, Gijón MA, Hankin JA (2011). MALDI imaging MS of phospholipids in the mouse lung. J Lipid Res.

[9] Murphy RC, Hankin JA, Barkley RM (2009). Imaging of lipid species by MALDI mass spectrometry. J Lipid Res.

[10] Brunelle A, Touboul D, Laprévote O (2005). Biological tissue imaging with time-of-flight secondary ion mass spectrometry and cluster ion sources. J Mass Spectrom.

[11] Todd PJ, Schaaff TG, Chaurand P, Caprioli RM (2001). Organic ion imaging of biological tissue with secondary ion mass spectrometry and matrix-assisted laser desorption/ionization. J Mass Spectrom.

[12] Touboul D, Brunelle A, Laprévote O (2011). Mass spectrometry imaging: towards a lipid microscope?. Biochimie.

[13] Chughtai K, Heeren RMA (2010). Mass spectrometric imaging for biomedical tissue analysis. Chem Rev.

[14] Kaletaş BK, Van Der Wiel IM, Stauber J, Dekker LJ, Güzel C, Kros JM (2009). Sample preparation issues for tissue imaging by imaging MS. Proteomics.

[15] Passarelli MK, Wang J, Mohammadi AS, Trouillon R, Gilmore I, Ewing AG (2014). Development of an organic lateral resolution test device for imaging mass spectrometry. Anal Chem.

[16] Peterson DS (2007). Matrix-free methods for laser desorption/ionization mass spectrometry. Mass Spectrom Rev.

[17] Xu S, Li Y, Zou H, Qiu J, Guo Z, Guo B (2003). Carbon nanotubes as assisted matrix for laser desorption/ionization time-of-flight mass spectrometry. Anal Chem.

[18] Tholey A, Heinzle E (2006). Ionic (liquid) matrices for matrix-assisted laser desorption/ionization mass spectrometry-applications and perspectives. Anal Bioanal Chem.

[19] Ayorinde F, Hambright P, Porter T, Keith Q (1999). Use of Meso-tetrakis(pentafluorophenyl)porphyrin as a matrix for low molecular weight alkylphenol ethoxylates in laser desorption/ ionization time-of-flight mass spectrometry. Rapid Commun Mass Spectrom.

[20] Guo Z, Zhang Q, Zou H, Guo B, Ni J (2002). A method for the analysis of low-mass molecules by MALDI-TOF mass spectrometry. Anal Chem.

[21] McCombie G, Knochenmuss R (2004). Small-molecule MALDI using the matrix suppression effect to reduce or eliminate matrix background interferences. Anal Chem.

[22] Stewart MP, Buriak JM (2000). Chemical and biological applications of porous silicon technology. Adv Mater.

[23] Rainer M, Qureshi MN, Bonn GK (2011). Matrix-free and material-enhanced laser desorption/ionization mass spectrometry for the analysis of low molecular weight compounds. Anal Bioanal Chem.

[24] Hayasaka T, Goto-Inoue N, Zaima N, Shrivas K, Kashiwagi Y, Yamamoto M (2010). Imaging mass spectrometry with silver nanoparticles reveals the distribution of fatty acids in mouse retinal sections. J Am Soc Mass Spectrom.

[25] Wen X, Dagan S, Wysocki VH (2007). Small-molecule analysis with silicon-nanoparticle-assisted laser desorption/ionization mass spectrometry. Anal Chem.

[26] Passarelli MK, Winograd N (2011). Lipid Imaging with time-of-flight secondary ion mass spectrometry (ToF-SIMS). Biochim Biophys Acta.

[27] Fletcher JS, Vickerman JC, Winograd N (2011). Label free biochemical 2D and 3D imaging using secondary ion mass spectrometry. Curr Opin Chem Biol.

[28] Toyoda N, Matsuo J, Aoki T, Yamada I, Fenner DB (2003). Secondary ion mass spectrometry with gas cluster ion beams. Appl Surf Sci.

[29] Rabbani S, Barber AM, Fletcher JS, Lockyer NP, Vickerman JC (2011). TOF-SIMS with argon gas cluster ion beams: a comparison with C60+. Anal Chem.

[30] Xu J, Ostrowski S, Szakal C, Ewing AG, Winograd N (2004). ToF-SIMS imaging with cluster ion beams. Appl Surf Sci.

[31] Tian H, Wucher A, Winograd N (2014). Molecular imaging of biological tissue using gas cluster ions. Surf Interface Anal.

[32] Angerer TB, Blenkinsopp P, Fletcher JS (2014). High energy gas cluster ions for organic and biological analysis by time-of-flight secondary Ion mass spectrometry. Int J Mass Spectrom.

[33] Phan NTN, Fletcher JS, Ewing AG (2015). Lipid structural effects of oral administration of methylphenidate in drosophila brain by secondary ion mass spectrometry imaging. Anal Chem.

[34] Jackson SN, Ugarov M, Egan T, Post JD, Langlais D, Schultz JA (2007). MALDI-ion mobility-TOFMS imaging of lipids in rat brain tissue. J Mass Spectrom.

[35] Mohammadi AS, Fletcher JS, Malmberg P, Ewing AG (2014). Gold and silver nanoparticle-assisted laser desorption ionization mass spectrometry compatible with secondary ion mass spectrometry for lipid analysis. Surf Interface Anal.

[36] Kimling J, Maier M, Okenve B, Kotaidis V, Ballot H, Plech A (2006). Turkevich method for gold nanoparticle synthesis revisited. J Phys Chem B.

[37] Phan NTN, Mohammadi AS, Dowlatshahi Pour M, Ewing AG (2016). Laser desorption ionization mass spectrometry imaging of drosophila brain using matrix sublimation versus modification with nanoparticles. Anal Chem.

[38] Fletcher JS, Rabbani S, Henderson A, Blenkinsopp P, Thompson SP, Lockyer NP (2008). A new dynamic in mass spectral imaging of single biological cells. Anal Chem.

[39] Hill R, Blenkinsopp P, Thompson S, Vickerman J, Fletcher JS (2011). A new time-of-flight SIMS instrument for 3D imaging and analysis. Surf Interface Anal.

[40] Angerer TB, Dowlatshahi MP, Malmberg P, Fletcher JS (2015). Improved molecular imaging in rodent brain with time-of-flight secondary ion mass spectrometry using gas cluster ion beams and reactive vapour exposure. Anal Chem.

[41] Altelaar AFM, Klinkert I, Jalink K, de Lange RPJ, Adan RAH, Heeren RMA (2006). Gold-enhanced biomolecular surface imaging of cells and tissue by SIMS and MALDI mass spectrometry. Anal Chem.

[42] Heile A, Lipinsky D, Wehbe N, Delcorte A, Bertrand P, Felten A (2008). Metal-assisted SIMS and cluster ion bombardment for ion yield enhancement. Appl Surf Sci.

[43] Delcorte A, Bour J, Aubriet F, Muller JF, Bertrand P (2003). Sample metallization for performance improvement in desorption/ionization of kilodalton molecules: quantitative evaluation, imaging secondary ion ms, and laser ablation. Anal Chem.

[44] Taira S, Sugiura Y, Moritake S, Shimma S, Ichiyanagi Y, Setou M (2008). Nanoparticle-assisted laser desorption/ionization based mass imaging with cellular resolution. Anal Chem.

[45] Murphy RC, Hankin JA, Barkley RM, Zemski Berry KA (2011). MALDI imaging of lipids after matrix sublimation/deposition. Biochim Biophys Acta Mol Cell Biol Lipids.

[46] Zavalin A, Todd EM, Rawhouser PD, Yang J, Norris JL, Caprioli RM (2012). Direct imaging of single cells and tissue at sub-cellular spatial resolution using transmission geometry MALDI MS. J Mass Spectrom.

[47] Novikov A, Caroff M, Della-Negra S, Lebeyec Y, Pautrat M, Schultz JA (2004). Matrix-implanted laser desorption/ionization mass spectrometry. Anal Chem.

[48] Tempez A, Ugarov M, Egan T, Schultz JA, Novikov A, Della-Negra S (2005). Matrix implanted laser desorption ionization (MILDI) combined with ion mobility-mass spectrometry for bio-surface analysis. J Proteome Res.

[49] Liu Y, Miao Z, Lakshmanan R, Loo RRO, Loo JA, Chen H (2012). Signal and charge enhancement for protein analysis by liquid chromatography–mass spectrometry with desorption electrospray ionization. Int J Mass Spectrom.

[50] Rao Ch S, Subash YE (2013). The effect of chronic tobacco smoking and chewing on the lipid profile. J Clin Diagn Res.

[51] Martínez-Beamonte R, Lou-Bonafonte JM, Martínez-Gracia MV, Osada J (2013). Sphingomyelin in high-density lipoproteins: structural role and biological function. Int J Mol Sci.

[52] Siegel GJ, Agranoff BW (1999). Basic neurochemistry: molecular, cellular and medical aspects.

[53] Kawahara K, Hohjoh H, Inazumi T, Tsuchiya S, Sugimoto Y (2015). Prostaglandin E2-induced inflammation: relevance of prostaglandin E receptors. Biochim Biophys Acta.

[54] Barreto-Bergter E, Pinto MR, Rodrigues ML (2004). Structure and biological functions of fungal cerebrosides. An Acad Bras Cienc.

[55] Messner MC, Cabot MC (2010). Glucosylceramide in humans. Adv Exp Med Biol.

[56] Vielhaber G, Pfeiffer S, Brade L, Lindner B, Goldmann T, Vollmer E (2001). Localization of ceramide and glucosylceramide in human epidermis by Immunogold electron microscopy. J Invest Dermatol.

[57] Podbielska M, Levery SB, Hogan EL (2011). The structural and functional role of myelin fast-migrating cerebrosides: pathological importance in multiple sclerosis. Clin Lipidol.

[58] Coetzee T, Fujita N, Dupree J, Shi R, Blight A, Suzuki K (1996). Myelination in the absence of Galactocerebroside and sulfatide: normal structure with abnormal function and regional instability. Cell.

[59] Dyer CA, Benjamins JA (1990). Glycolipids and transmembrane signaling: antibodies to galactocerebroside cause an influx of calcium in oligodendrocytes. J Cell Biol.

[60] Adibhatla RM, Hatcher JF (2007). Role of lipids in brain injury and diseases. Future Lipidol.

[61] Kaddurah-Daouk R, McEvoy J, Baillie R, Zhu H, Yao KJ, Nimgaonkar VL (2012). Impaired plasmalogens in patients with schizophrenia. Psychiatry Res.

[62] Mikawa S, Suzuki M, Fujimoto C, Sato K (2009). Imaging of phosphatidylcholines in the adult rat brain using MALDI-TOF MS. Neurosci Lett.

[63] Carter CL, McLeod CW, Bunch J (2011). Imaging of phospholipids in formalin fixed rat brain sections by matrix assisted laser desorption/ionization mass spectrometry. J Am Soc Mass Spectrom.

[64] Cerruti CD, Benabdellah F, Laprévote O, Touboul D, Brunelle A (2012). MALDI imaging and structural analysis of rat brain lipid negative ions with 9-aminoacridine matrix. Anal Chem.

[65] Angel PM, Spraggins JM, Baldwin HS, Caprioli R (2012). Enhanced sensitivity for high spatial resolution lipid analysis by negative ion mode matrix assisted laser desorption ionization imaging mass spectrometry. Anal Chem.

[66] Son J, Lee G, Cha S (2014). Direct analysis of triacylglycerols from crude lipid mixtures by gold nanoparticle-assisted laser desorption/ionization mass spectrometry. J Am Soc Mass Spectrom.

