# Supplementary material for: Intact lipid imaging of mouse brain samples: MALDI, nanoparticle-laser desorption ionization, and 40 keV argon cluster secondary ion mass spectrometry
Source: Anal Bioanal Chem. 2016 Aug 22;408(24):6857–68. doi: 10.1007/s00216-016-9812-5 (PMC5012256; doi:10.1007/s00216-016-9812-5)
Supplement: Supplementary file 1 — (PDF 1.17 mb) [file 216_2016_9812_MOESM1_ESM.pdf]

Analytical and Bioanalytical Chemistry

Electronic Supplementary Material

Intact lipid imaging of mouse brain samples: MALDI, nanoparticle-laser desorption ionization, and 40 keV argon cluster secondary ion mass spectrometry

Amir Saeid Mohammadi, Nhu T.N. Phan, John S. Fletcher, Andrew G. Ewing

In this Supporting Information, we present figures demonstrating homogenous distribution of Au nanoparticles on mouse brain slice, compatibility of nanoparticle modified mouse brain sample with Ar cluster SIMS analysis and we showed that Ar cluster SIMS analysis prior to NP-LDI doesn't induce any chemical change on mouse brain sample.

Furthermore we showed a wide m/z range (150-900 Da) mass spectra of mouse brain obtained by 40 keV Ar_{4000}^+ SIMS, NP –LDI and MALDI DHB sublimation. Additionally low mass ion images for NP-LDI and over layer ion images for NP-LDI and MALDI are presented.

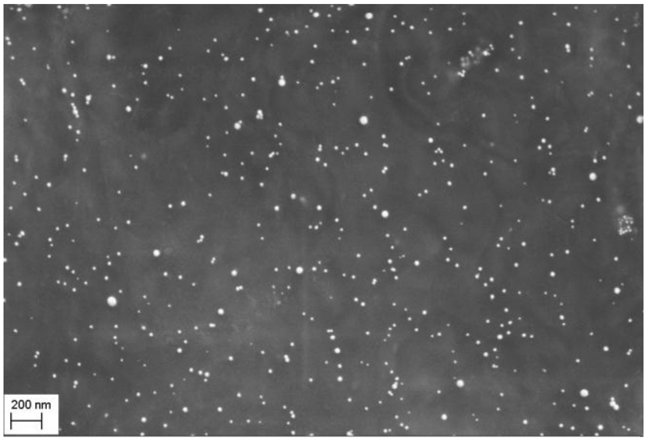

Fig. S1 Scanning electron microscopy (SEM) image of nanoparticle deposition on brain section. The SEM image shows the homogeneous deposition of sprayed 10 nm Au nanoparticles (bright dots) on top of the mouse brain tissue slice, which is critical in order to obtain good ionization and spatial resolution

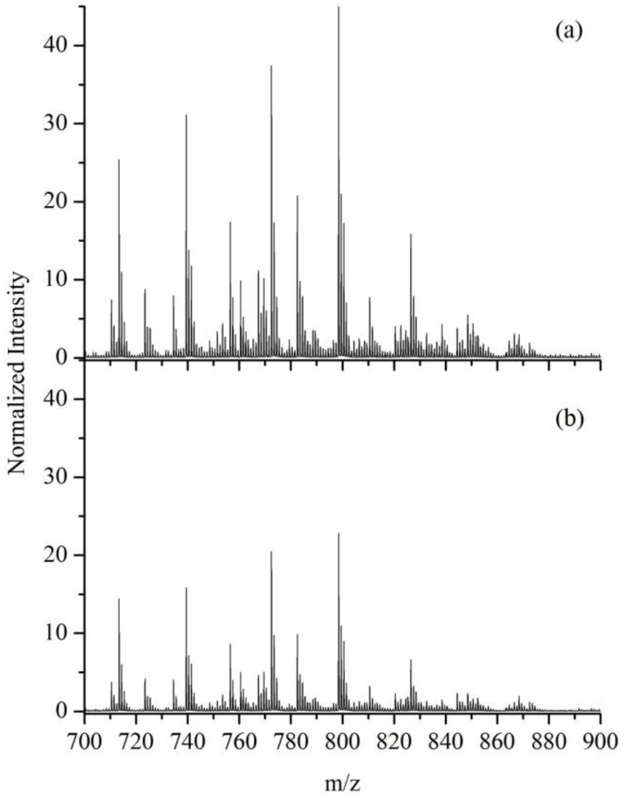

Fig. S2 Nanoparticle modified mouse brain sample showed compatibility with SIMS analysis. High energy Ar cluster SIMS spectrum with NPs sample modification (a) and without any modification (b). No chemical change was seen by NP modification. All spectra are normalized to the total number of selected pixels

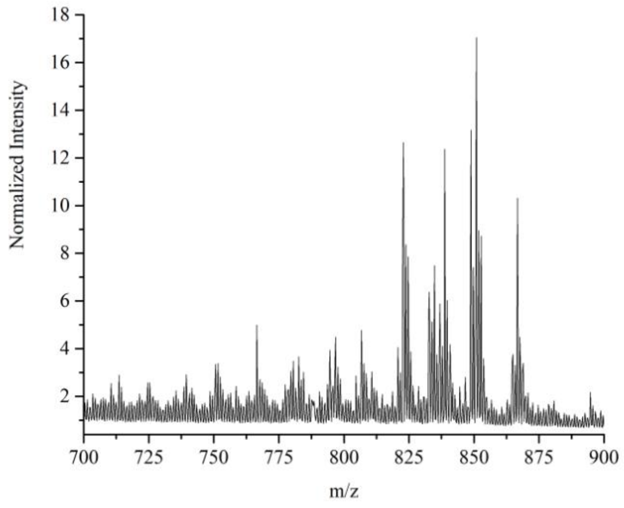

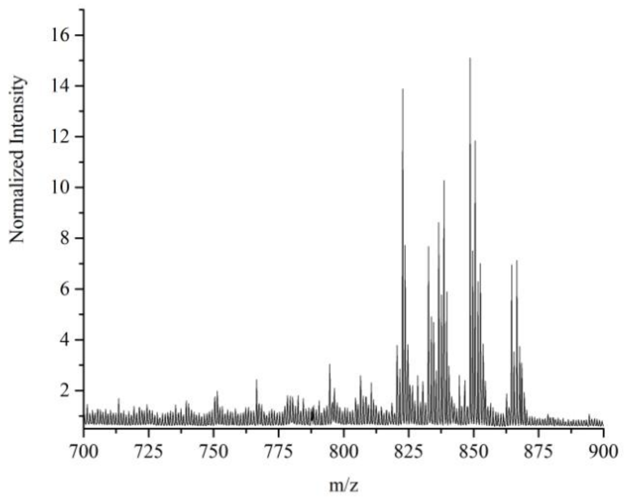

Fig. S3 Mass spectra of nanoparticle modified mouse brain obtained by NP-LDI after Ar cluster SIMS analysis (a) and a fresh nanoparticle modified mouse brain sample (without any prior SIMS analysis) (b). All spectra are normalized to the total number of selected pixels

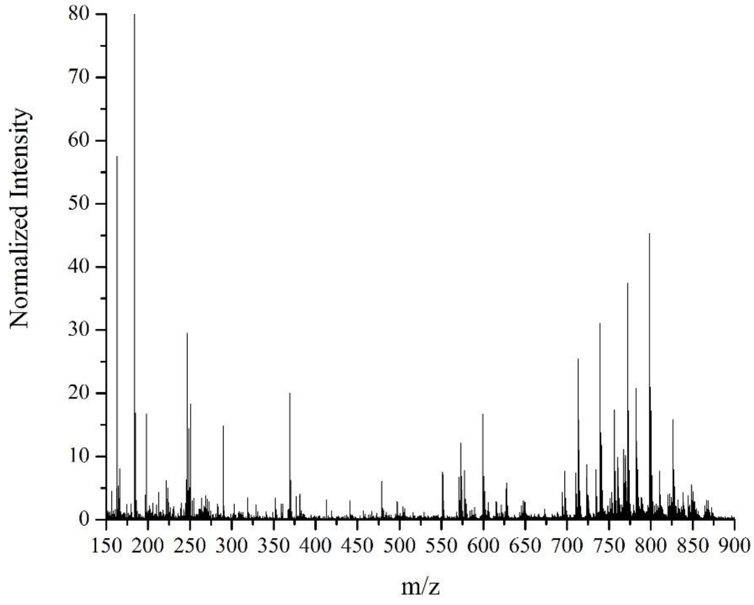

40 keV Ar_{4000}^{+} SIMS spetrum of a mouse brain slice

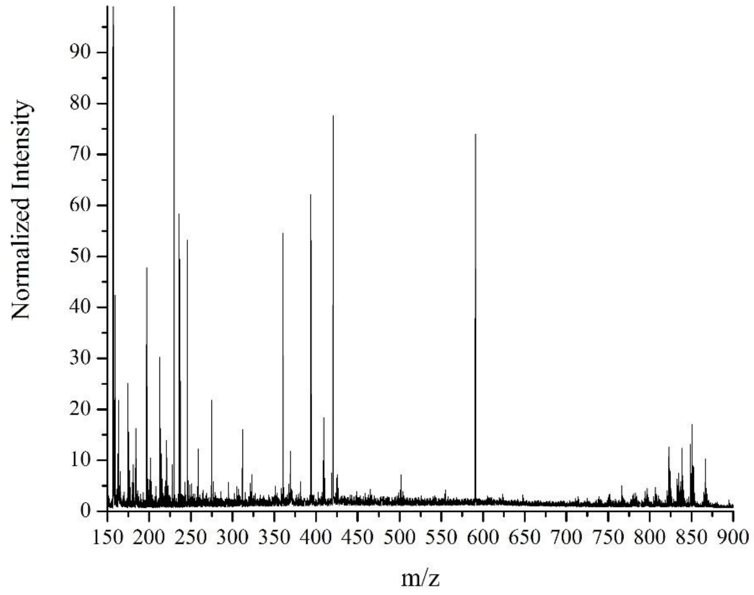

NP-LDI mass spectrum of a mouse brain slice

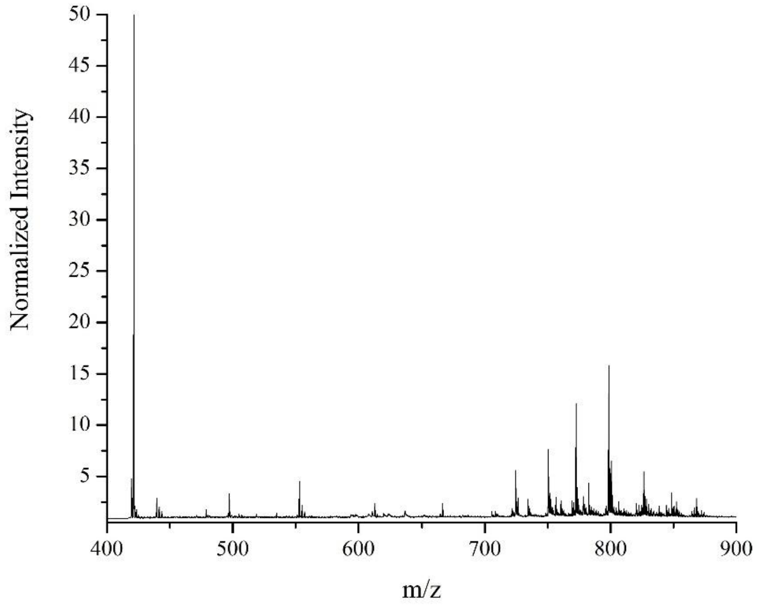

MALDI DHB sublimation mass spectrum of a mouse brain slice

Fig. S4 Mass spectra of a mouse brain sample (both grey and white matter) obtained by GCIB SIMS, NP-LDI and MALDI in a wide m/z range (150-900 Da). Spectrum obtained by SIMS method is normalized to the total number of selected pixels. Spectra obtained by MALDI and NP-LDI are normalized to total ion counts

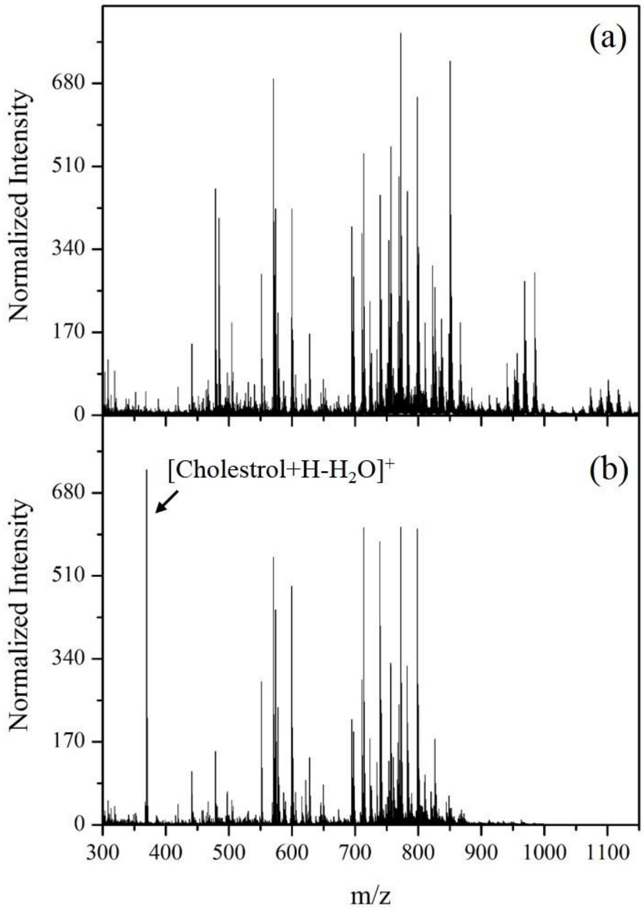

Fig. S5 Mass spectra of a mouse brain sample showing the cholestrol content peak in control sample (b) and TFA exposure sample (a) showing the diminution of cholestrol in the white matter after TFA exposure. All spectra are normalized to the total number of selected pixels

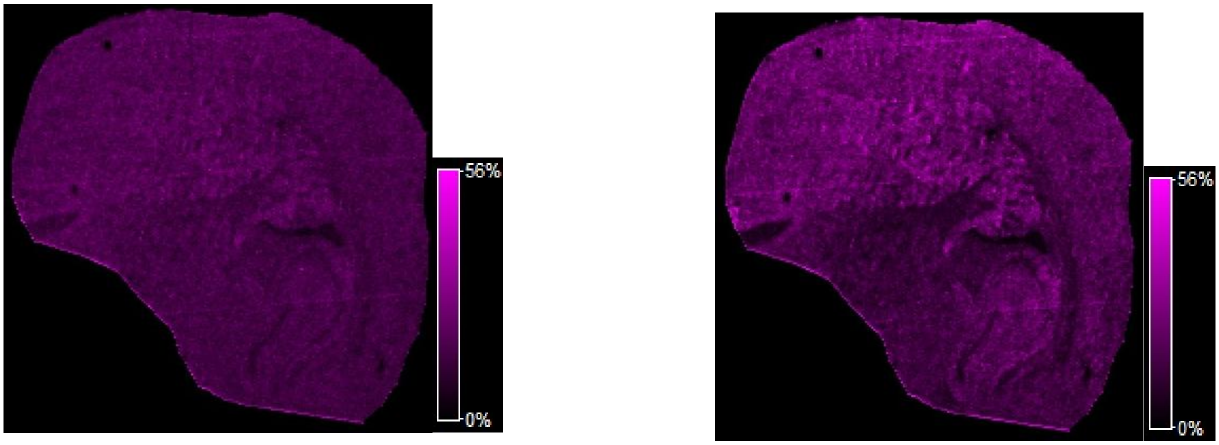

m/z 734 **m/z 772**

Fig. S6 Ion images for DHB MADLI showing the distribution of PC (32:0) at m/z 734 $[M+H]^+$ and m/z 772 $[M+K]^+$

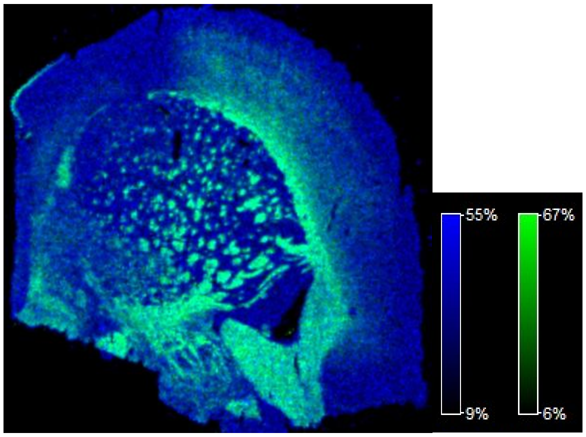

(A)

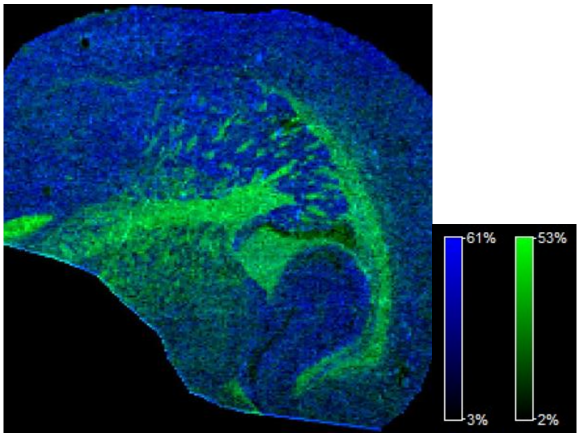

(B)

Fig. S7 Over layer ion images (total ion counts normalization) for NP-LDI (A) and MALDI (DHB sublimation) (B) to show the lipids distribution in the white matter (m/z 850.6, green) and grey matter (772.6, blue). (4800 × 4800 μm^2)

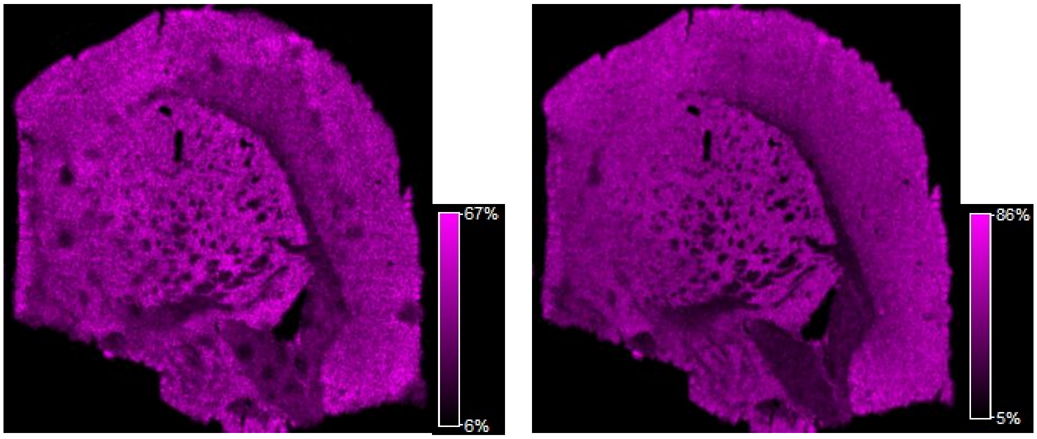

m/z 184.0 m/z 86.1

Fig. S8 Ion images (4800×4800 μm^2) of low mass fragments obtained by NP-LDI (PC lipid low mass fragments)
